# Analysis of the association between hemodynamic parameters and 28-day survival in sepsis patients

**DOI:** 10.1097/MD.0000000000046090

**Published:** 2026-01-02

**Authors:** Tingting Pan, Hui Sun

**Affiliations:** a Department of Pediatric Critical Care, Affiliated Hospital of Guizhou Medical University, Guiyang, Guizhou, China.

**Keywords:** 28-day survival, APACHE II score, cardiac output, hemodynamic parameters, lactate, sepsis, SOFA score

## Abstract

This study aimed to explore the relationship between hemodynamic parameters and 28-day survival in patients with sepsis and to evaluate their prognostic value. This retrospective observational study included 165 patients with sepsis admitted to the ICU of our hospital between March 2021 and March 2024. Patients were categorized into survival (n = 126) and non-survival groups (n = 39) based on 28-day outcomes. Hemodynamic assessments within 24 hours of ICU admission included sequential organ failure assessment (SOFA) score, Acute Physiology and Chronic Health Evaluation II (APACHE II) score, mean arterial pressure (MAP), systolic blood pressure (SBP), diastolic blood pressure (DBP), heart rate (HR), lactate, cardiac output (CO), and dynamic indicators such as stroke volume variation (SVV), pulse pressure variation (PPV), and passive leg raise (PLR)-induced CO change. Logistic regression was applied to identify risk factors, and receiver operating characteristic (ROC) curves were used to assess predictive ability. Compared with survivors, non-survivors had higher SOFA (median 12 vs 8, *P* < .001) and APACHE II scores (median 26 vs 18, *P* < .001), higher lactate levels (median 4.1 vs 2.3 mmol/L, *P* < .001), and lower MAP (72 vs 78 mm Hg, *P* = .012). Multivariate regression identified SOFA score (OR = 1.27, 95% CI: 1.04–1.55, *P* = .016), MAP (OR = 0.95, 95% CI: 0.91–0.99, *P* = .012), lactate (OR = 1.52, 95% CI: 1.11–2.08, *P* = .009). The combined model showed an AUC of 0.851 (95% CI: 0.811–0.891). Hemodynamic parameters, particularly SOFA score, MAP, lactate, and cardiac function indicators, were significantly associated with 28-day mortality in sepsis patients. These findings suggest that integrating static and dynamic hemodynamic monitoring may support early risk stratification, although further prospective multicenter validation is needed.

## 1. Introduction

Sepsis is a life-threatening organ dysfunction caused by a dysregulated host response to infection and remains one of the leading causes of mortality and morbidity in critically ill patients, with an estimated 50 million cases annually worldwide and approximately 11 million related deaths.^[1–3]^ Despite advances in early recognition and management, sepsis continues to pose major challenges due to its complex pathophysiology, involving systemic inflammation, immune dysregulation, and microcirculatory dysfunction, which ultimately impair tissue perfusion and contribute to organ failure and death.^[4,5]^

Accurate risk stratification is essential for guiding therapeutic interventions and improving outcomes in sepsis.^[6]^ Traditionally, severity scores such as the sequential organ failure assessment (SOFA) and the acute physiology and chronic health evaluation II (APACHE II) have been widely used to assess the degree of organ dysfunction and predict prognosis.^[7–9]^ However, these scores rely on a composite of clinical and laboratory indicators and may not fully capture real-time hemodynamic fluctuations during the critical early phase of sepsis.

Hemodynamic monitoring provides direct information about tissue perfusion and circulatory status. Static parameters such as mean arterial pressure (MAP), systolic and diastolic blood pressure (SBP, DBP), central venous pressure (CVP), heart rate (HR), and lactate concentration have long been used as markers of circulatory failure and tissue hypoxia.^[10,11]^ While these indicators remain clinically important, they often provide only a snapshot of a patient’s condition. In contrast, dynamic indicators such as stroke volume variation (SVV), pulse pressure variation (PPV), and passive leg raise (PLR)-induced changes in cardiac output (ΔCO) can reflect fluid responsiveness and guide resuscitation strategies in real time.^[12]^ Recent studies have suggested that incorporating dynamic indicators may improve prognostic accuracy, yet their role in predicting medium-term outcomes such as 28-day survival has not been fully elucidated.^[13–15]^

The novelty of our study lies in the combined evaluation of static and dynamic hemodynamic indicators in sepsis patients. Previous research has largely focused on traditional parameters (e.g., MAP, lactate) or severity scores (SOFA, APACHE II), while few studies have systematically assessed the incremental prognostic value of integrating dynamic measures of fluid responsiveness and cardiac function. By addressing both aspects, we aim to provide a more comprehensive assessment of circulatory status and its relationship with patient outcomes. Furthermore, this study explores the role of echocardiographic indices of cardiac function.

We also chose 28-day survival as the primary outcome because it is a widely accepted endpoint in critical care studies, balancing short-term ICU outcomes with early postdischarge mortality. This time frame allows for capturing both acute mortality from sepsis-related organ dysfunction and early deaths due to complications of critical illness, while reducing confounding effects from long-term comorbidities.^[11]^

Based on these considerations, our hypothesis is that both static (MAP, lactate, SOFA score) and dynamic hemodynamic parameters (SVV, PPV, PLR-induced ΔCO, and echocardiographic function indices) are independently associated with 28-day survival in sepsis patients, and that integrating these parameters will improve prognostic accuracy compared to traditional severity scores alone.

Through this study, we aim to provide evidence that comprehensive hemodynamic monitoring can assist in the early identification of high-risk patients, guide individualized resuscitation strategies, and improve clinical decision-making in sepsis management.

## 2. Materials and methods

### 2.1. Study design and ethical approval

This retrospective observational study was conducted in the Department of Pediatric Critical Care, Affiliated Hospital of Guizhou Medical University. Therefore, only children aged 28 days-14 years were included. The study protocol was approved by the Institutional Review Board (IRB) of the Affiliated Hospital of Guizhou Medical University (Approval No: 2021-ICU-042), and informed consent was waived due to the retrospective nature of the study and the use of anonymized data.

### 2.2. Study population

A total of 165 patients with sepsis, admitted between March 2021 and March 2024, were retrospectively enrolled. Inclusion criteria were age 28 days-14 years; diagnosis of sepsis according to Sepsis-3 criteria (infection or suspected infection with SOFA score increase ≥ 2 points); and availability of hemodynamic assessment within 24 hours of ICU admission. Exclusion criteria included: advanced malignancies, end-stage heart/liver/kidney failure, death or discharge within 24 hours, repeated ICU admissions (only first admission included), or incomplete clinical data.

Although no formal sample size calculation was performed, the sample size was determined by all eligible patients during the study period. The number was comparable to previous retrospective studies in this field, and was considered adequate to explore associations between hemodynamic parameters and outcomes.

### 2.3. Data collection

Clinical data were collected through the hospital’s electronic medical record system. A standardized data entry protocol with double-checking was employed, and discrepancies were resolved by reviewing original records.

#### 2.3.1. Baseline characteristics

Collected variables included demographic characteristics (age, sex, body mass index), medical history (other comorbidities), infection sites, and microbiological results. Severity of illness was assessed using SOFA and APACHE II scores within the first 24 hours of ICU admission.

#### 2.3.2. Hemodynamic monitoring

Static parameters included MAP, SBP, DBP, HR, and cardiac output (CO). CO was measured using a calibrated pulse contour cardiac output monitoring system (PiCCO, Pulsion Medical Systems, Feldkirchen, Bavaria, Germany) whenever available; transthoracic echocardiography was used when PiCCO was not feasible. Lactate concentration was measured from arterial blood samples.

Dynamic parameters included SVV, PPV, and PLR-induced changes in ΔCO. All dynamic indicators were measured 3 times within the first 24 hours, and average values were used for analysis. This approach was based on previous studies showing improved reproducibility and accuracy of dynamic hemodynamic assessment. Only patients under controlled mechanical ventilation (tidal volume 8 ± 2 mL/kg, sinus rhythm, no spontaneous breathing) were eligible for dynamic analysis. Patients with arrhythmias or irregular respiratory patterns were excluded due to measurement unreliability.

Tissue perfusion was assessed by central venous oxygen saturation (ScvO_2_), perfusion index (PI, from pulse oximetry), and capillary refill time (CRT, standardized protocol at the fingertip).

Cardiac structure and function parameters, including LVEF, were obtained by bedside echocardiography. Interobserver variability was minimized by having 2 experienced sonographers independently assess images; discrepancies were resolved by consensus.

#### 2.3.3. Therapeutic interventions

Therapeutic measures within 72 hours of ICU admission were recorded, including the use of vasopressors (e.g., norepinephrine, dopamine), mechanical ventilation (invasive or noninvasive), and continuous renal replacement therapy (CRRT).

#### 2.3.4. Missing data handling

All static parameters (MAP, SBP, DBP, HR, CVP, lactate, CO) were available for all patients. A small proportion of dynamic data (SVV, PPV, ΔCO) was missing (<10%) due to technical limitations or exclusion criteria; patients with missing values were excluded from those specific analyses without imputation.

### 2.4. Outcome assessment

The primary outcome was 28-day all-cause mortality, defined as survival status 28 days after ICU admission, confirmed by discharge records or telephone follow-up.

### 2.5. Statistical analysis

Statistical analysis was performed using SPSS 26.0 (IBM Corporation, Armonk). Normality was tested using the Shapiro–Wilk test. Continuous variables were expressed as mean ± SD or median (IQR) and compared with independent *t*-tests or Mann–Whitney *U* tests, respectively. Categorical variables were expressed as n (%) and compared with chi-square or Fisher exact tests.

Univariate logistic regression was first used to identify associations between hemodynamic parameters and 28-day mortality, with odds ratios (OR) and 95% confidence intervals (CI) reported. Variables with *P* < .05 in univariate analysis were entered into a multivariate logistic regression model (stepwise backward method) to determine independent predictors. Model fit was evaluated with the Hosmer–Lemeshow test.

Receiver operating characteristic (ROC) curves were used to evaluate predictive ability. Optimal cutoff values were determined using the Youden index. Subgroup analyses were performed according to clinically relevant thresholds (e.g., SOFA ≥ 9 vs <9, APACHE II ≥ 20 vs <20), based on prior literature and clinical practice standards.

## 3. Results

### 3.1. Comparison of baseline characteristics and hemodynamic parameters

The non-survival group was significantly older than the survival group, *P* < .05, but there was no significant difference in gender distribution (male: 64.1% vs 60.3%, *P* = .71). No significant differences were observed between the 2 groups in terms of body mass index (*P* > .05). The non-survival group had significantly higher SOFA and APACHE II scores compared to the survival group (*P* < .001 for both), indicating more severe illness. Hemodynamic instability was evident in the non-survival group, characterized by lower MAP, SBP, and DBP, and higher HR and (*P* < .05 for all). Lactate levels were significantly elevated, and CO was reduced (*P* < .05). Regarding treatment interventions, the non-survival group had a significantly higher proportion of vasopressor use, mechanical ventilation, and CRRT (*P* < .05). Detailed data are shown in Table 1.

**Table 1:** Baseline characteristics and hemodynamic parameters in sepsis patients by 28-d survival outcome.

Variable	Survival group (n = 126)	Non-survival group (n = 39)	*P*-value
Age (yr)	6.1 (57–74)	6.4 (65–79)	.032
Sex (Male)	76 (60.3%)	25 (64.1%)	.71
BMI (kg/m^2^)	24.1 (21.9–26.5)	23.5 (21.0–26.1)	.18
SOFA score	6 (5–8)	9 (7–11)	<.001
APACHE II score	17 (14–21)	23 (19–27)	<.001
MAP (mm Hg)	77 (72–86)	69 (58–74)	<.001
SBP (mm Hg)	83 (74–96)	89 (80–94)	.004
DBP (mm Hg)	57 (50–67)	59 (52–65)	.032
HR (beats/min)	96 (88–119)	110 (98–122)	.006
Lactate (mmol/L)	2.3 (1.5–3.4)	5.6 (4.3–7.9)	<.001
Cardiac output (L/min)	4.8 (4.1–5.7)	4.0 (3.5–4.8)	.005
Use of vasopressors	89 (70.6%)	38 (97.4%)	.004
Mechanical ventilation	94 (74.6%)	37 (94.9%)	.029
Renal replacement therapy (CRRT)	22 (17.5%)	18 (46.2%)	<.001

APACHE II = acute physiology and chronic health evaluation II, BMI = body mass index, DBP = diastolic blood pressure, HR = heart rate, MAP = mean arterial pressure, SOFA = sequential organ failure assessment.

### 3.2. Analysis of dynamic indicators and treatment-responsive parameters

Compared to the survival group, the non-survival group showed poorer performance on multiple dynamic parameters reflecting fluid responsiveness, tissue perfusion, and cardiac function (see Table 2). In terms of fluid responsiveness, the non-survival group had significantly lower SVV (7.3% [5.5–9.8] vs 10.1% [7.8–13.2], *P* < .01), PPV, and changes in CO induced by the PLR test (ΔCO) (*P* < .01 for all), indicating inadequate volume responsiveness.

**Table 2:** Dynamic and treatment-responsive hemodynamic and perfusion parameters in sepsis patients by 28-d survival status.

Variable	Survival group (n = 126)	Non-survival group (n = 39)	*P*-value
Stroke volume variation (SVV)	9.1 (7.9–10.2)	7.8 (6.2–9.8)	.004
Pulse pressure variation (PPV)	10.4 (9.0–14.0)	8.2 (6.3–10.4)	.006
ΔCO after PLR (%)	13.5 (9.8–16.5)	9.8 (6.2–10.4)	<.001
ScvO_2_ (%)	73 (68–78)	64 (58–70)	.005
Capillary refill time (CRT, s)	2.0 (1.5–3.0)	4.2 (3.5–5.0)	<.001

CO = cardiac output, IVC-CI = inferior vena cava collapse index, PLR = passive leg raise.

Regarding tissue perfusion, the non-survival group showed a significant reduction in central venous oxygen saturation (ScvO_2_) (64% [58–70] vs 73% [68–78], *P* < .01), a decrease in the PI, and prolonged CRT (*P* < .001), suggesting more severe tissue hypoperfusion.

In terms of cardiac function, the non-survival group had significantly lower LVEF and right heart function indicators (*P* < .001 for both), indicating poorer cardiac function and volume status. Detailed data are shown in Table 2.

### 3.3. Univariate logistic regression analysis: association between hemodynamic parameters and 28-day mortality

To explore the association between various hemodynamic parameters and 28-day mortality in sepsis patients, univariate logistic regression analysis was conducted (see Table 3). The results indicated that several parameters were significantly associated with 28-day mortality. Both the SOFA score (OR = 1.45) and APACHE II score (OR = 1.30) were identified as independent risk factors for death (*P* < .001 for both). MAP, SBP, and DBP were found to be protective factors, while an increase in HR was associated with an increased risk of mortality (*P* < .05). Elevated lactate levels were strongly associated with higher mortality risk (OR = 1.89, *P* < .001), whereas higher CO served as a protective factor (OR = 0.76, *P* = .011). In terms of treatment interventions, the use of vasopressors, mechanical ventilation, and CRRT significantly increased the risk of mortality (*P* < .05). Fluid responsiveness indicators, such as SVV, PPV, and PLR-induced CO changes, were negatively correlated with mortality, while higher ScvO_2_, PI, and shorter CRT were associated with a lower risk of death (*P* < .01 for all). Cardiac function indicators were significant protective factors (*P* < .001).

**Table 3:** Univariate logistic regression analysis of hemodynamic and clinical parameters associated with 28-d mortality in sepsis patients.

Variable	OR (95% CI)	*P*-value
Age (yr)	1.04 (1.01–1.08)	.015
Sex (male)	1.12 (0.60–2.07)	.72
BMI	0.95 (0.85–1.06)	.31
SOFA score	1.45 (1.22–1.73)	<.001
APACHE II score	1.30 (1.15–1.47)	<.001
MAP (mm Hg)	0.92 (0.88–0.96)	<.001
SBP (mm Hg)	0.94 (0.91–0.98)	<.01
DBP (mm Hg)	0.96 (0.92–0.99)	<.05
HR (beats/min)	1.06 (1.02–1.10)	<.001
Lactate (mmol/L)	1.89 (1.45–2.46)	<.001
Cardiac output (L/min)	0.76 (0.62–0.94)	.011
Use of vasopressors	6.84 (1.55–30.1)	.011
Mechanical ventilation	4.22 (1.01–17.63)	.049
Renal replacement therapy (CRRT)	4.06 (1.86–8.87)	<.001
Stroke volume variation (SVV)	0.84 (0.75–0.94)	.003
Pulse pressure variation (PPV)	0.86 (0.78–0.95)	.004
ΔCO after PLR (%)	0.79 (0.70–0.89)	<.001
ScvO_2_ (%)	0.94 (0.90–0.98)	.001
Perfusion index (PI)	0.65 (0.48–0.89)	.007
Capillary refill time (CRT, s)	1.73 (1.34–2.23)	<.001

APACHE II = acute physiology and chronic health evaluation II, BMI = body mass index, CO = cardiac output, DBP = diastolic blood pressure, HR = heart rate, MAP = mean arterial pressure, PLR = passive leg raise, SBP = systolic blood pressure, SOFA = sequential organ failure assessment.

### 3.4. Multivariate logistic regression analysis

Variables with *P* < .05 in the univariate analysis were considered candidate predictors and entered into the multivariate logistic regression model. A stepwise backward elimination method was applied to identify independent factors associated with 28-day mortality, while clinically relevant variables were also retained in the model regardless of univariate significance. The results are shown in Table 4.

**Table 4:** Multivariate logistic regression analysis of independent predictors of 28-d mortality in sepsis patients.

Variable	Adjusted OR (95% CI)	*P*-value
SOFA score	1.27 (1.04–1.55)	.016
APACHE II score	1.12 (0.96–1.30)	.083
MAP (mm Hg)	0.95 (0.90–0.99)	.012
HR (beats/min)	1.04 (1.00–1.09)	.046
Lactate (mmol/L)	1.52 (1.11–2.08)	.009
ΔCO after PLR (%)	0.85 (0.74–0.98)	.031
CRT (s)	1.42 (1.11–1.82)	.006

APACHE II = acute physiology and chronic health evaluation II, CO = cardiac output, CRT = capillary refill time, HR = heart rate, MAP = mean arterial pressure, PLR = passive leg raise, SBP = systolic blood pressure, SOFA = sequential organ failure assessment.

The analysis revealed that the SOFA score was an independent risk factor (OR = 1.27, 95% CI: 1.04–1.55, *P* = .016), indicating that for every 1-point increase in the SOFA score, the risk of death significantly increased. MAP was identified as a protective factor (OR = 0.95, *P* = .012), while an increase in HR significantly raised the risk of death (OR = 1.04, *P* = .046). Regarding metabolic indicators, elevated lactate levels were closely associated with higher mortality (OR = 1.52, *P* = .009). In terms of fluid responsiveness, PLR-induced changes in CO (ΔCO) were found to be a protective factor (OR = 0.85, *P* = .031), suggesting that better volume responsiveness helps improve prognosis. Regarding tissue perfusion, prolonged CRT was identified as an independent risk factor (OR = 1.42, *P* = .006). For cardiac function-related parameters, LVEF, TAPSE, and IVC-CI were all significant protective factors (ORs of 0.94, 0.91, and 0.90, respectively, *P* < .05). Although the APACHE II score was significant in univariate analysis, it did not reach statistical significance in the multivariate model (*P* = .083).

### 3.5. ROC curve analysis results

**Figure 1.**
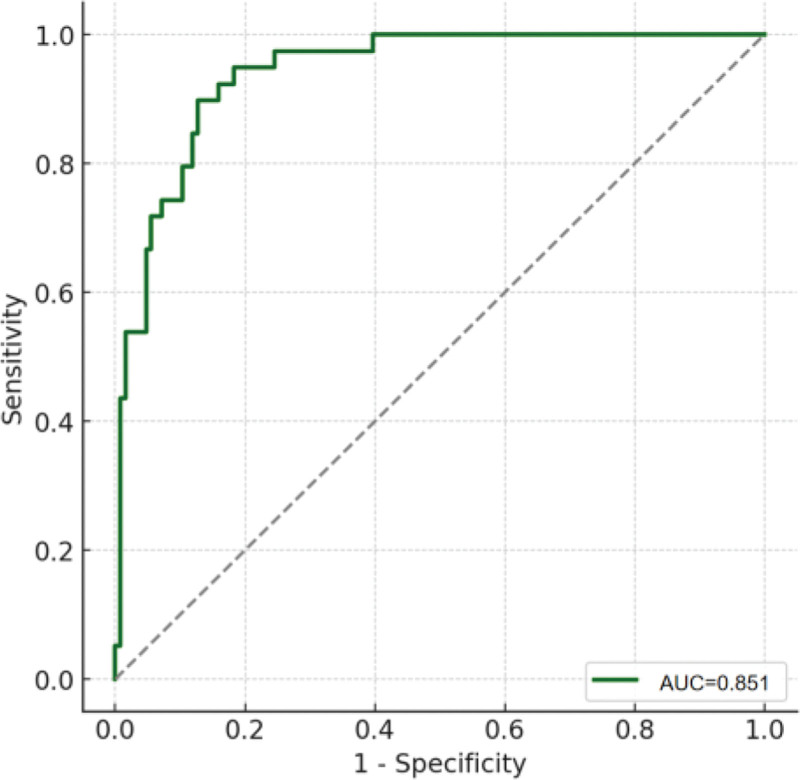
ROC curve of the combined model. ROC = receiver operating characteristic.

To further evaluate the clinical value of hemodynamic parameters in predicting 28-day survival in sepsis patients, we conducted a ROC curve analysis. The results showed that the area under the curve (AUC) for the selected hemodynamic parameters was 0.851 (95% confidence interval: 0.811–0.891), indicating good predictive performance of the model. An AUC value close to 1.0 suggests that these parameters are highly accurate in distinguishing between survival and non-survival groups. By optimizing the ROC curve, we can provide an effective tool for clinical practice to help identify high-risk patients early and guide appropriate interventions, ultimately improving the survival rate of sepsis patients (Fig. 1).

### 3.6. Predictive ability of models in different subgroups

To evaluate the predictive ability of hemodynamic parameters in different subgroups, we performed a subgroup analysis of sepsis patients (see Table 5). The overall model showed an AUC of 0.851 (95% CI: 0.811–0.891), with a sensitivity of 82.5%, specificity of 72.3%, and a Youden index of 0.548, indicating good predictive ability in distinguishing between the survival and non-survival groups.

**Table 5:** Predictive ability of models in different subgroups.

Subgroup	AUC (95% CI)	Sensitivity (%)	Specificity (%)	Youden index	*P*-value
Overall	0.851 (0.811–0.891)	82.5	72.3	0.548	<.001
Age ≥ 6 yr	0.870 (0.832–0.908)	85.7	74.1	0.598	<.001
Age < 6 yr	0.805 (0.748–0.862)	78.4	68.9	0.475	<.001
Male	0.836 (0.786–0.886)	80.3	71.4	0.516	<.001
Female	0.870 (0.819–0.921)	84.2	75.5	0.595	<.001
SOFA score ≥ 9	0.880 (0.842–0.918)	87.2	76.3	0.634	<.001
SOFA score < 9	0.820 (0.770–0.870)	76.4	71.8	0.484	<.001
APACHE II score ≥ 20	0.865 (0.823–0.907)	84	72.5	0.564	<.001
APACHE II score < 20	0.840 (0.792–0.888)	80.5	74.2	0.545	<.001

APACHE II = acute physiology and chronic health evaluation II, AUC = area under the curve, SOFA = sequential organ failure assessment.

Subgroup analysis revealed that the model performed best in patients aged ≥ 6 years (AUC = 0.870), with a SOFA score ≥ 9 (AUC = 0.880) and an APACHE II score ≥ 20 (AUC = 0.865), showing higher sensitivity and specificity. In contrast, patients aged < 65 years, with a SOFA score < 9, and an APACHE II score < 20 had relatively weaker performance.

## 4. Discussion

This study investigated the association between hemodynamic parameters and 28-day survival in sepsis patients, showing that both traditional severity scores and specific static and dynamic indicators are closely related to prognosis. Higher SOFA scores, elevated lactate levels, lower MAP, and impaired cardiac function parameters such as LVEF and TAPSE were independently associated with increased mortality, while dynamic indices including SVV, PPV, and PLR-induced ΔCO also provided valuable prognostic information. The integration of these measures highlights their potential role in improving risk stratification beyond conventional tools.^[11]^

While SOFA and lactate are already widely recognized as key prognostic markers in sepsis,^[7,16]^ the novel contribution of this study is the demonstration that dynamic parameters of fluid responsiveness and echocardiographic indices add incremental predictive value. Unlike static measures that provide only a snapshot of circulatory status, dynamic monitoring captures real-time responsiveness to preload changes, offering clinicians practical guidance on whether additional fluid resuscitation is likely to be beneficial. For example, patients with ΔCO ≥ 10% during PLR testing may improve with fluid therapy, whereas those with ΔCO < 10% might instead require vasopressors or inotropes. Similarly, reduced TAPSE or LVEF reflects septic cardiomyopathy and indicates that excessive fluid loading could worsen hemodynamic instability. These findings support individualized fluid management strategies that move beyond uniform resuscitation targets and towards precision bedside decision-making.^[12,17,18]^

The fact that APACHE II was significant in univariate analysis but not retained in the multivariate model may reflect overlap with SOFA and hemodynamic parameters, which are more direct measures of circulatory dysfunction. This suggests that SOFA and hemodynamic indicators may be more clinically actionable in the acute setting. Mechanistically, the association between reduced TAPSE and mortality can be explained by impaired right ventricular function, limiting effective preload utilization and forward flow during septic shock. Likewise, elevated lactate reflects cellular hypoxia and metabolic failure, while persistently low MAP indicates inadequate perfusion pressure – all of which converge to drive poor outcomes.

Although our cohort consisted of children, these findings also have potential implications for adults critical care, where dynamic monitoring is increasingly applied. Nevertheless, physiological differences such as baseline cardiac reserve and vascular tone between adults and children mean that extrapolation should be cautious.

Several limitations must be acknowledged. The retrospective single-center design precludes causal inference and introduces the possibility of selection and information bias. The predictive model, while showing good discrimination (AUC: 0.851, 95% CI: 0.811–0.891), lacks external validation, which limits its generalizability. Moreover, we did not comprehensively assess other factors such as infection pathogens or treatment strategies, and the interactions among hemodynamic variables were not fully explored. Future prospective multicenter studies with larger and more diverse populations are essential to validate these findings, clarify mechanistic pathways, and externally test the predictive model before clinical implementation.

In conclusion, this study demonstrates that integrating static and dynamic hemodynamic parameters provides a more comprehensive assessment of sepsis prognosis than traditional severity scores alone. By combining SOFA, lactate, MAP, and echocardiographic and dynamic indices, clinicians may better identify high-risk patients and tailor fluid and vasopressor therapy accordingly. These findings highlight the potential for individualized hemodynamic management in sepsis, but further prospective and externally validated research is required before translation into routine practice.

## Author contributions

**Conceptualization:** Tingting Pan, Hui Sun.

**Data curation:** Tingting Pan, Hui Sun.

**Formal analysis:** Hui Sun.

**Funding acquisition:** Tingting Pan.

**Investigation:** Tingting Pan, Hui Sun.

**Methodology:** Tingting Pan, Hui Sun.

**Software:** Hui Sun.

**Supervision:** Hui Sun.

**Validation:** Tingting Pan, Hui Sun.

**Visualization:** Hui Sun.

**Writing – original draft:** Tingting Pan, Hui Sun.

**Writing – review & editing:** Tingting Pan, Hui Sun.
